# Influence of increased injected activities up 9.0 GBq [^177^Lu]Lu-PSMA-I&T on absorbed doses to lesions and kidneys

**DOI:** 10.1186/s40658-026-00932-x

**Published:** 2026-09-11

**Authors:** Zachary Ells, Linus Hempel, Julia Ells, Muyu Wu, Maximilian Scheifele, Magdalena Schoell, Sophie C. Siegmund, Mathias Zacherl, Rudolf A. Werner, Jeremie Calais, Magnus Dahlbom, Guido Böning, Adrien Holzgreve, Astrid Delker, Lena M. Unterrainer

**Affiliations:** 1 Department of Nuclear Medicine, LMU University Hospital, LMU Munich, Marchioninistr. 15, 81377 Munich, Germany; 2 Department of Nuclear Medicine and Theranostics, Ahmanson Translational Theranostics Division, David Geffen School of Medicine at UCLA, University of California Los Angeles UCLA, Los Angeles, CA USA; 3 Bavarian Cancer Research Center (BZKF), Munich, Germany; 4 German Cancer Consortium (DKTK), Partner Site Munich, A Partnership Between DKFZ and LMU University Hospital Munich, Munich, Germany

**Keywords:** Lutetium, Metastatic castration-resistant prostate cancer (mCRPC), Prostate-specific membrane antigen, Radiopharmaceutical therapy, Tumor sink effect

## Abstract

**Introduction:**

Administration activities of ^177^Lu-PSMA were chosen cautiously in the treatment of advanced prostate cancer. The primary aim was to investigate the association between increased injected activities, and the calculated absorbed doses (AD).

**Methods:**

In this single center, retrospective analysis, patients with PSMA-avid metastatic castration-resistant prostate cancer treated with [^177^Lu]Lu-PSMA-I&T were included. Patients underwent multiple timepoint SPECT/CT imaging at 24, 48, and 72 h post-injection. Patients included were injected with 7.4, or 9.1 GBq. Segmentations were completed manually for the kidneys (CT-based), while a 3.0 SUV threshold-based approach was used for lesions. Dosimetry calculations were then performed using a multiple-timepoint protocol. Manual verification of the individual segmentations coregistered between timepoints was completed. All time activity data was fit using a mono-exponential function. Time-integrated activity was then convolved with voxel-s-value using a predefined ^177^Lu kernel resulting the AD in Gy. No further partial volume corrections were made.

**Results:**

76 patients treated were included: 22 and 54 were injected with 9.1 ± 0.3 and 7.4 ± 0.3 GBq respectively. The AD/GBq to the kidney was comparable, 0.29 ± 0.08 vs. 0.33 ± 0.13 Gy/GBq [*p*=0.193] (9.1 vs. 7.4 GBq). The tumor AD/GBq was comparable between 9.1 and 7.4 GBq cohorts: total tumor burden (0.95 ± 0.42 vs. 0.92 ± 0.52 Gy/GBq) [*p*=0.302]; bone lesions 0.78 ± 0.42 vs. 0.74 ± 0.46 Gy/GBq [*p*=<0.001]; hottest lesion in each patient 1.37 ± 0.61 vs. 1.30 ± 0.74 [*p*=0.421]; coldest lesion in each patient 0.40 ± 0.16 vs. 0.50 ± 0.32 Gy/GBq [*p*=0.930].

**Conclusion:**

The AD/GBq across the disease burden, individual lesions, and kidney remained comparable even with increased injected activity. No evidence of the tumor sink effect was found. No evidence of a clinically relevant tumor sink effect in patients with <2000 mL tumor volume was found but patients with substantially high TTV (> 2 L) lacks.

**Supplementary Information:**

The online version contains supplementary material available at https://doi.org/10.1186/s40658-026-00932-x.

## Introduction

Prostate cancer is a common diagnosis in men [1], often characterized by the overexpression of prostate specific membrane antigen (PSMA) on cancer cells [2] making it a key target in imaging and therapy. The effectiveness of ^177^Lu-PSMA compounds in the treatment of metastatic castration-resistant prostate cancer has been shown in both prospective single-arm [3–5], and randomized [6–8] clinical trials.

After the VISION Trial conclusion, the therapy has been approved using an administered activity of 7.4 GBq every 6 weeks. This time interval selected was based on known hematologic safety considerations (blood count nadir at 3 to 6 weeks after molecular radiopharmaceutical therapy (RPT) administration) reported in the randomized prospective phase 3 trials [9–11]. The 7.4 GBq activity regimens were chosen based on dosimetry data [12, 13] and the NETTER-1 trial experience [10].

In the absence of organ-specific tolerance data for RPT, the administered activity has historically been limited by absorbed dose (AD) constraints derived from external beam radiation therapy (EBRT) [14]. Specifically, organ AD limits such as 18–23 Gy for kidneys were adopted from the EBRT tolerance data compiled [15, 16]. Using the average dose deposition of 4.7 Gy/[^177^Lu]Lu-PSMA-I&T-cycle from published data this would indicate only 3 or 4 cycles could be delivered before reaching such thresholds. Therefore these AD limits are potentially overly conservative due to the low-dose rate exposure from molecular RPT compared to high dose rate of EBRT. Higher activity regimens were safely reported in the German compassionate use studies (up to 9.7 GBq/cycle) [17] and the Australian clinical trials (up to 8.7 GBq/cycle) [4, 6, 18]). Of note in the phase 1 dose-escalation study NCT03042468, up to 22.2 GBq/cycle was safely administered with promising early efficacy and tolerability signals [19].

Nevertheless, a recent publication [20] parsed 95 international theranostic centers and found that the mean standard injected activity for ^177^Lu-PSMA based therapy was 7.3 GBq/cycle (range 5.5–11.1 GBq). In a recent meta-analysis the AD to tumors did not change with the injected activity [21] (range 3.6–7.5 GBq) across numerous publications. Direct comparison of the reported AD to tumors from various studies however are limited due to lack of standardization in imaging protocols, lesion segmentation method employed, and AD calculation which all affect the reported estimates [22, 23].

In RPT, increasing the injected activity is motivated by increasing the number of decays occurring within tumor tissue, thereby increasing the AD. There is growing clinical evidence, specifically for ^177^Lu-PSMA therapies, showing that higher cumulative, or per-cycle activities, are associated with increased tumor AD and improved response rates [24–27].

Herein the primary aim of this study was to investigate if the AD estimates to tumors and kidneys change based upon a 23% increase in the injected activity (7.4 vs. 9.1 GBq).

## Methods

### Study design and patient population

**Fig. 1:**
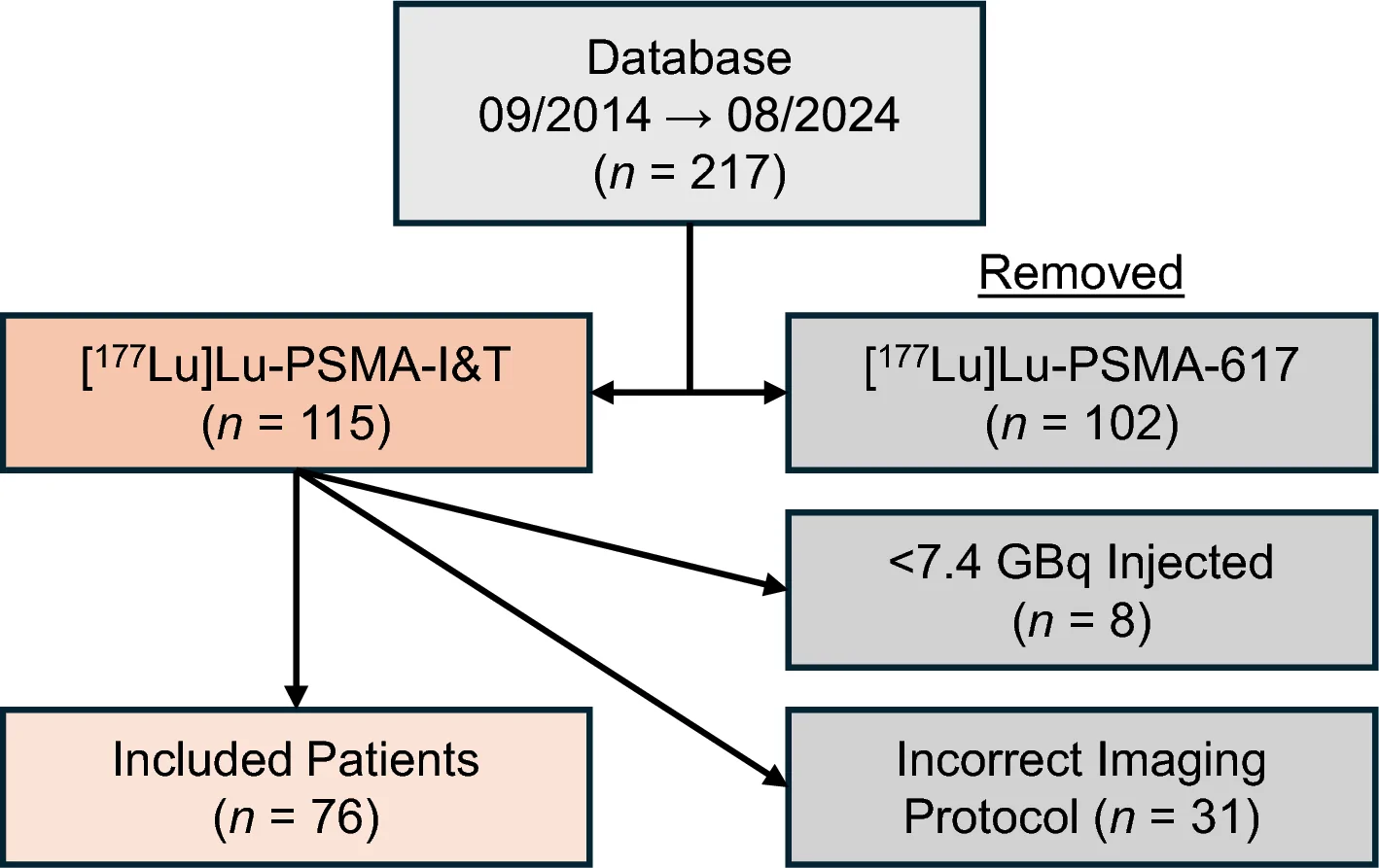
Patient flowchart of included patients

In this single center retrospective analysis, patients with PSMA-avid metastatic castration-resistant prostate cancer treated with [^177^Lu]Lu-PSMA-I&T were included (Fig. 1). Patients included must have had multiple timepoint SPECT/CT imaging at 24, 48, and 72 h post-injection while having no prior RPT. PSA values were obtained upon patient follow-up to the clinic four weeks after the first cycle.

Two sub-cohorts of patients were created, the first injected with 7.4 ± 0.1 GBq, and the second with 9.1 ± 0.3 GBq. The difference in specific activity was negligible between the 7.4 GBq and 9.1 GBq sub-cohorts; prepared therapies used 200 and 250 µg of PSMA-I&T precursor, respectively, corresponding to specific activities of approximately 0.037 and 0.036 GBq/µg.

### Imaging protocol

Detailed information of the patient acquisition protocol can be found as previously described [28]. Briefly, quantitative SPECT images were acquired using the Siemens Symbia (Siemens Healthineers, Erlangen, Germany) (upper photopeak of 208 keV, width: 15%). An acquisition protocol consisted of three bed positions, 64 projections per detector head measured with 5 s measurement time per projection. Images were acquired using a matrix of 128 *× * 128 pixels with size of (4.8 mm)^2^. CareDose was enabled using a reference current of 15 mAs, and tube voltage of 110–130 kV.

The raw data was reconstructed using Hermes Hybrid Recon-Oncology 4.0 (Hermes Medical Solutions, Stockholm, Sweden). The reconstruction protocol used a maximum-a-posteriori ordered-subset expectation–maximization (MAP-OSEM; β=0.001) algorithm with 16 iterations and 8 subsets [29]. Quantitative reconstruction included CT-based attenuation correction, Monte Carlo-based scatter correction, and resolution modeling [30].

### Segmentation

The single click segmentation tool available in HERMES Affinity (version 3.0.5) was used to segment each individual lesion using an SUV threshold of 3.0 as suggested by John et al. on the 24 h post-injection timepoint [31]. The total tumor volume (TTV) therefore was determined by summation of all individual tumor volumes. The functional unit of the kidney (e.g. cortex, and medulla) were segmented manually on the 24 h post-injection timepoint CT.

### Dosimetry estimates

Tumor and kidney ADs were based on multiple timepoint SPECT at 24, 48, and 72 h post-injection after the first cycle of therapy. Dosimetry calculations were performed using MIM SurePlan™ MRT (MIM Software Inc., Cleveland, Ohio, USA; version 7.3.4). Manual verification of SPECTs coregistered between timepoints were completed for each individual segmentation. Time activity data for each volume of interest were fit using a mono-exponential function. Time-integrated activity was then convolved with voxel-s-value using a predefined ^177^Lu kernel resulting the AD in Gy. No spill-over correction was applied. Lesions below 3 mL in volume, and rib lesions were removed from the AD calculation due to partial volume effects [32, 33] as well as coregistration and motion errors during imaging.

### Total tumor volume analysis

The total tumor volume (TTV) represents the entire disease burden present in the patient. To systematically evaluate between the two injected activities a sub-analysis stratifying the TTV into distinct ranges (100–380 mL, and 500–2000 mL) was completed. The tumor sink effect was assessed. The AD/GBq over the entire tumor volume (AD_TTV_) removed lesions prone high uncertainty as previously mentioned. The AD_TTV_ is representative of the average AD/GBq deposition over the total segmented lesion volume per the MIM Software protocol.

### Statistical analysis

Correlations between the two injected activities were calculated and compared. All analyses were conducted using GraphPad Prism (Boston, Massachusetts, USA; version 8.4.3). Welch’s *t*-tests were performed to determine statistical significance, assuming a Gaussian distribution for unpaired data. *P*-values less than 0.05 were considered statistically significant. All values are represented as median [range] or average ± standard deviation unless otherwise specified.

## Results

### Patient characteristics

A total of 76 patients were included in the study (Fig. 1), 54 were injected with 7.4 ± 0.1 GBq and 22 were injected with 9.1 ± 0.3 GBq [*p* = <0.0001]. The increased injected activities administered were determined by expert physician based on patient specific characteristics. The patient characteristics including demographics of prior treatments, and risk factors associated with [^177^Lu]Lu-PSMA-I&T administration are supplied in Table 1.

**Table 1:** Patient characteristics

Characteristic	7.4 GBq	9.1 GBq	*P*—value
Patients Included	54	22	
Age (years)	75 [53–89]	74 [55–86]	0.870
Baseline PSA (ng/mL)	72 [0.4–2845]	262 [8–1696]	0.051
Post-Cycle 1 Follow-Up PSA (ng/mL)	65 [0.7–2412]	264 [12–3808]	0.055
Prior PCa-directed Treatments*:			
Abiraterone	31	16	
Enzalutamide	35	14	
Docetaxel	40	17	
Cabazitaxel	21	6	
^223^Radium	1	0	
Leuprorelin	19	9	
Other†	26	13	
Undefined ADT‡	13	3	
*Risk Factors:*			
Diabetes Mellitus (D.M)	6	2	
Arterial Hypertension (A.H)	11	4	
Renal Disease (R.D)	1	0	
D.M + A.H	4	2	
D.M + A.H + R.D	1	0	
*Patient Disease Characteristic:*			
Injected Activity [GBq]	7.4 ± 0.1	9.1 ± 0.3	<0.0001
Median Injected Acitivity [GBq]	7.4	9.0	
SPECT/CT TTV [mL]	368.9 ± 521.2	1164.5 ± 1157.0	<0.0001
Lesions Per Patient	7 [1–21]	10 [3–21]	
Total Number of Lesions	405	228	
Bone Lesions	330	183	
Lymph Node Lesions	71	43	
Liver Lesions	4	2	

^*^In two patients prior treatments were unable to be determined

^†^Other includes: Darolutamid, Bicalutamid, Apalutamid, Denosumab, Olaparib, Zoledronat, Ipilimumab, Nivolumab, Prednisolon, Triptorelin, Flutamid, Carboplatin,

^‡^Undefined ADT: Undefined or secondary treatment of ADT

**Fig. 2:**
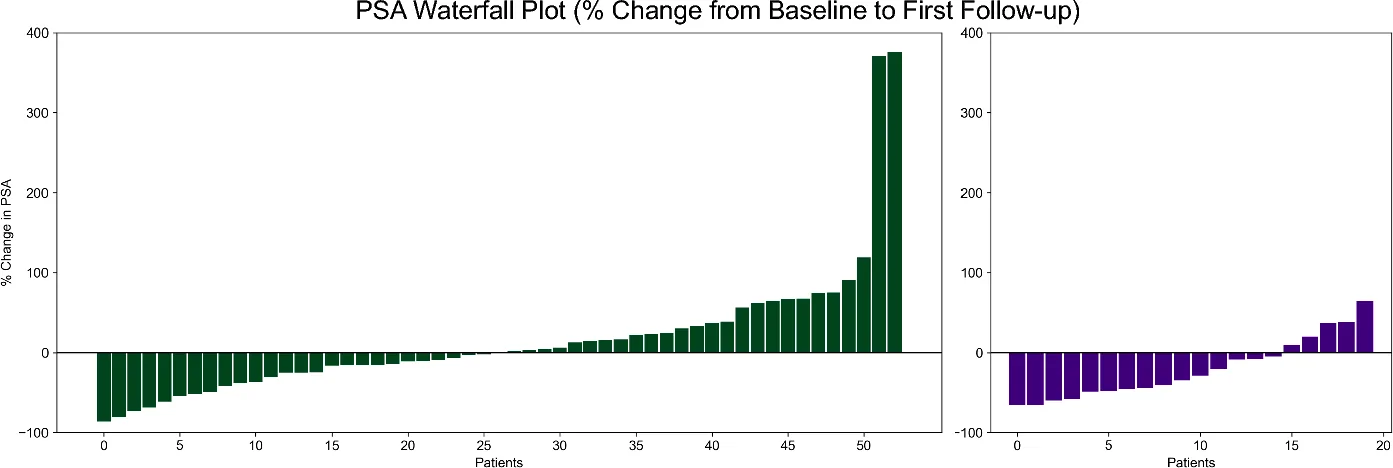
(Left) 7.4 vs. (Right) 9.1 GBq. 7/53 (13%) and 4/20 (20%) achieved a PSA≥50% decline

Clinical evaluation of PSA in 3 patients were not possible. 1/54 (2%) and 2/22 (10%) receiving 7.4 and 9.1 GBq respectively. All removed patients had non-specific PSA values recorded (i.e. “> 1000 ng/dL”). A statistically significant difference in the percent change in PSA from baseline was observed between patients receiving 7.4 GBq (n = 53) to those receiving 9.1 GBq (n = 20); median change of −1% (− 86–371) and −32% (− 65–65), respectively [p = 0.024] (Fig. 2).

### Total tumor volume metrics

**Fig. 3:**
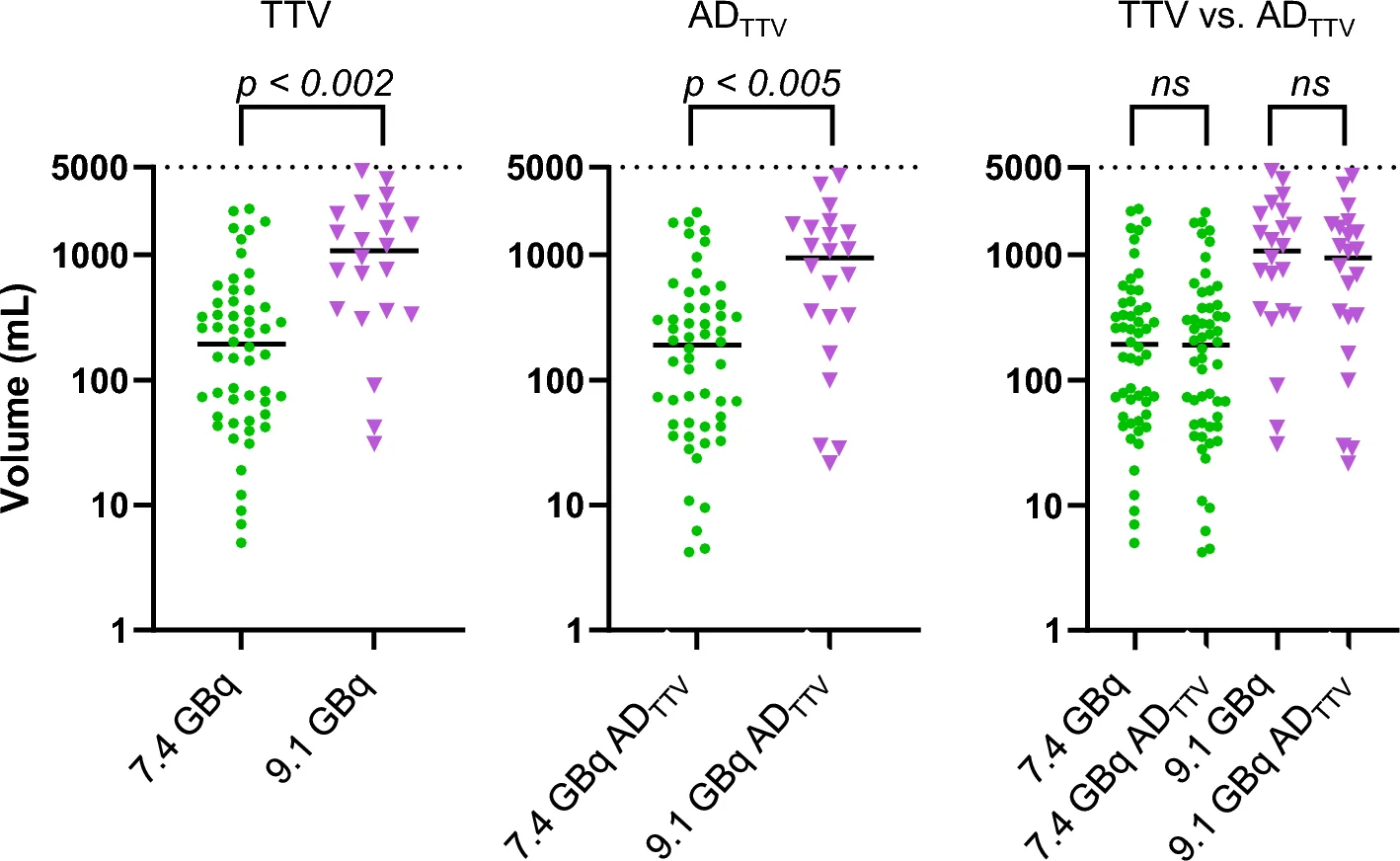
Distribution of patients based on TTV (left); the effects of removing lesions <3 mL and rib lesions (middle); comparison between TTV and AD_TTV_ between the two injected activity regimens (right)

The TTV between the 7.4 and 9.1 GBq cohorts were significantly different, 189.8 mL [4.2–2182.5] vs. 947.3 mL [21.7–4308.9] [*p* = <0.002] (Fig. 3 -Left). After removing high uncertainty lesions (represented as AD_TTV_) the difference between the two injected activity regimens were still significantly different [p = <0.005] (Fig. 3—Middle). The difference between the TTV and AD_TTV_ was not statistically significant in either injected activity regimens (Fig. 3—Right). This indicates only a small volume of disease was removed in the AD calculation (AD_TTV_). Supplemental Table 1 material shows the volume quantification TTV and AD_TTV_ in tabular format.

### Absorbed dose estimates to lesions

The AD/GBq were calculated across the entire tumor burden (AD_TTV_), and for each individual lesion stratified by anatomic location. As the two cohorts differed in TTV, a sub-analysis stratified by TTV was also performed. Patients were grouped into two categories for comparison: those with TTVs between 100–380 mL and 500–2000 mL. While the 100–380 mL group represented greater similarity between TTV [*p* = 0.839], the groups were uneven (19 patients injected with 7.4 GBq vs. 5 patients injected with 9.1 GBq). The 500–2000 mL group represented similar TTV [*p* = 0.689], but with an equal number of patients (*n* = 11).

**Fig. 4:**
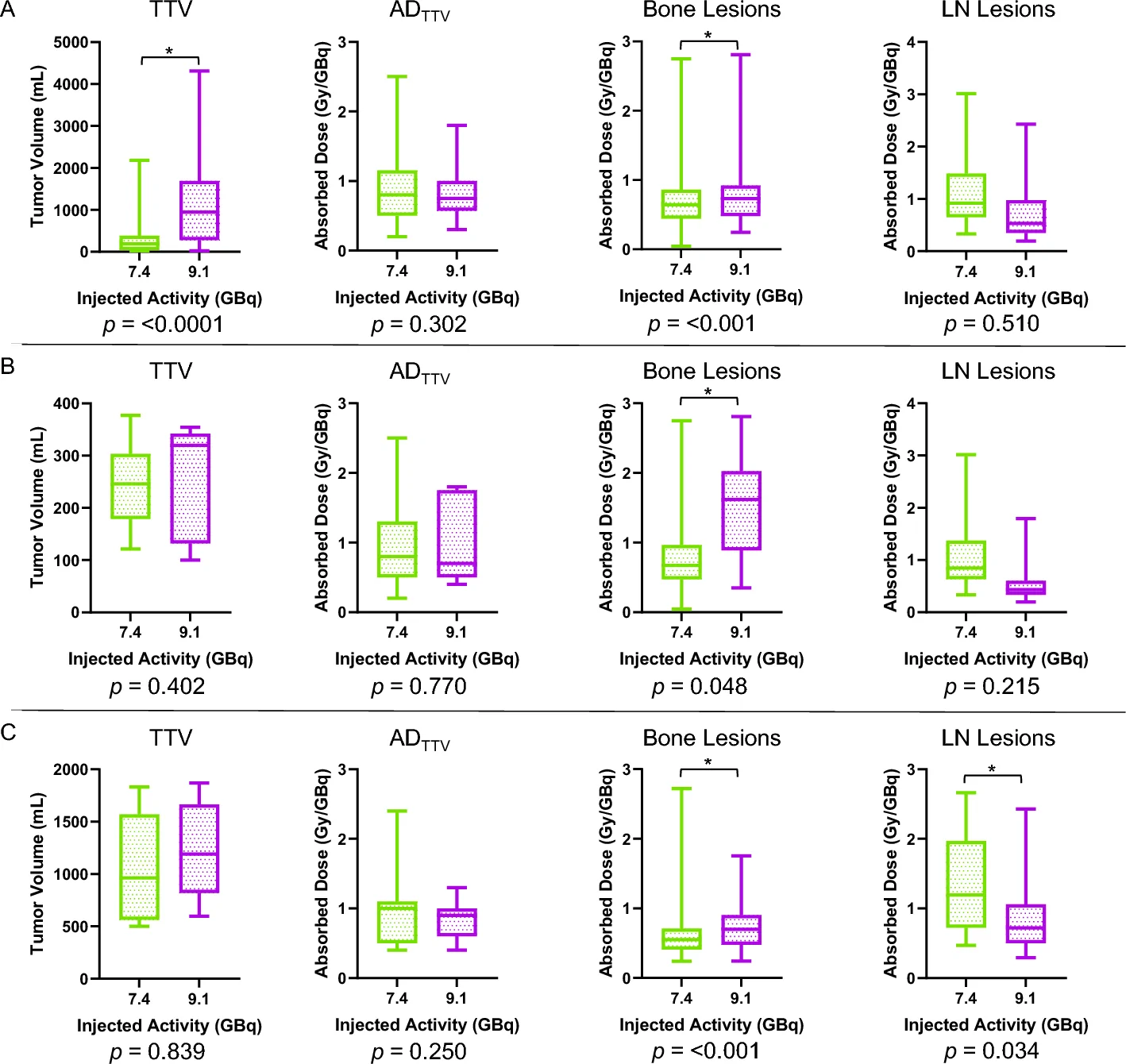
Comparison of the two injected activity regimens for whole TTV, and AD/GBq estimates over the whole disease burden (AD_TTV_), and within individual lesions based on anatomical location. **A** All patients in both cohorts. **B** Stratified by TTV 100–380 mL (*n* = 19 and 5 for 7.4 and 9.1 GBq respectively), **C** Stratified by TTV 500–2000 mL (*n* = 11 in both cohorts). Number of lesions can be found in Table 1

Figure 4 summarizes our findings of the AD/GBq across the total tumor burden (2nd column), and to the individual lesions (3rd and 4th column). AD_TTV_ tended to only be slightly greater in the 9.1 GBq cohort compared to 7.4 GBq. Additionally, in bone lesions a significantly higher [*p* = <0.001] AD/GBq was found in the 9.1 GBq cohort. Lymph node lesions support no difference in AD/GBq between the two injected activities.

**Fig. 5:**
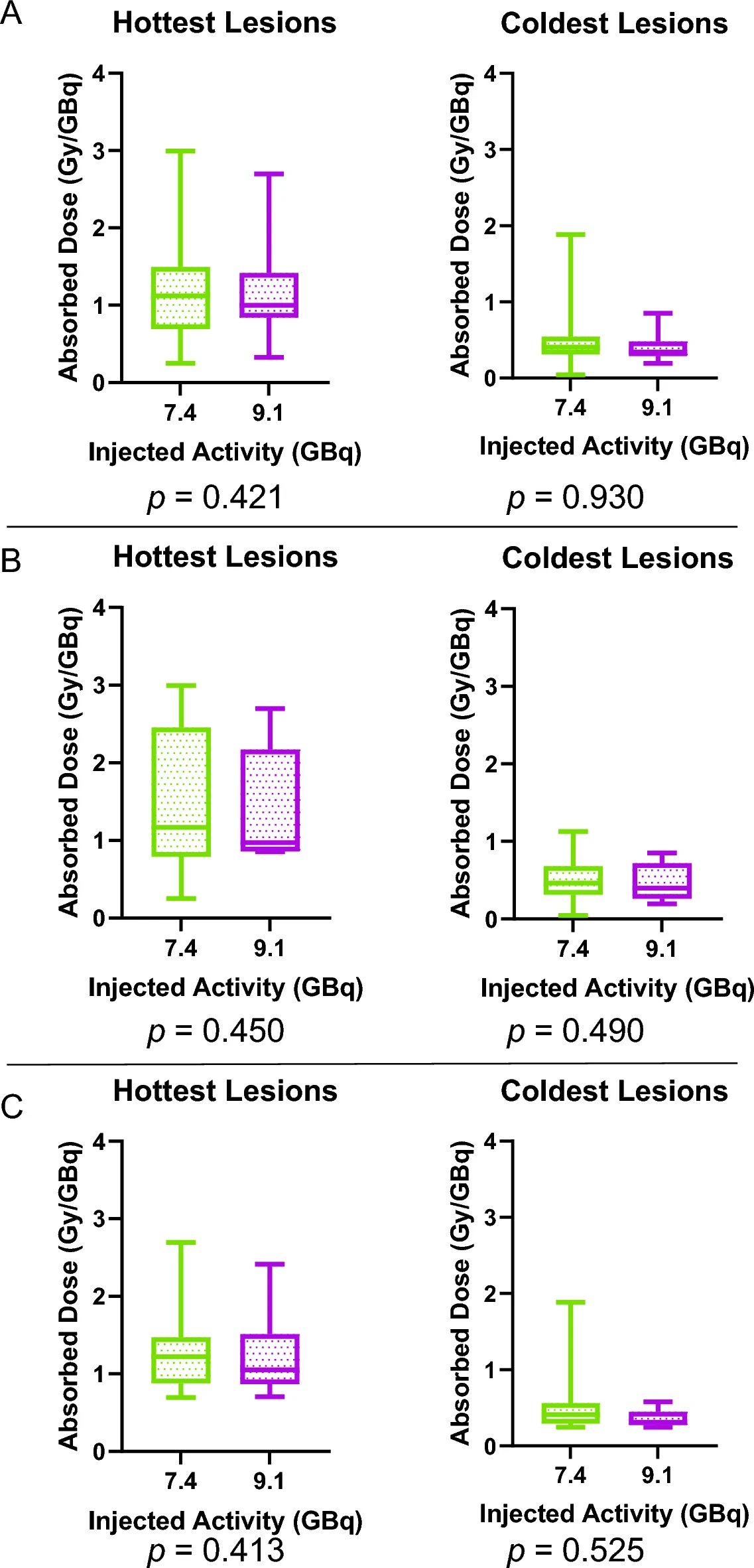
Comparison of the hottest and coldest lesion present in each patient treated by the two injection activities. **A** All patients in both cohorts. **B** Stratified by TTV 100–380 mL (*n* = 19 and 5 for 7.4 and 9.1 GBq respectively), **C** Stratified by TTV 500–2000 mL (*n* = 11 in both cohorts)

The hottest and coldest lesion present in each patient was investigated to identify if the increased injected activity influenced this aspect of treatment. There was no statistical significance between hottest and coldest lesion between the two different injection activities administered [*p* = 0.421 and 0.930] respectively. Figure 5 summarizes the findings across the two injected activities (top), and stratified by TTV (middle and bottom). Due to the applied segmentation method 7/54 patients (13%) in 7.4 GBq group and 6/22 patients (30%) in 9.1 GBq group were not able to have the hottest and coldest lesion evaluated as there was only a single lesion present. All individual lesions and AD_TTV_ for these patients were included in the rest of the analysis. Tabular format of the AD analysis can be found in the supplemental tables 3–6.

### Kidney absorbed dose

**Fig. 6:**
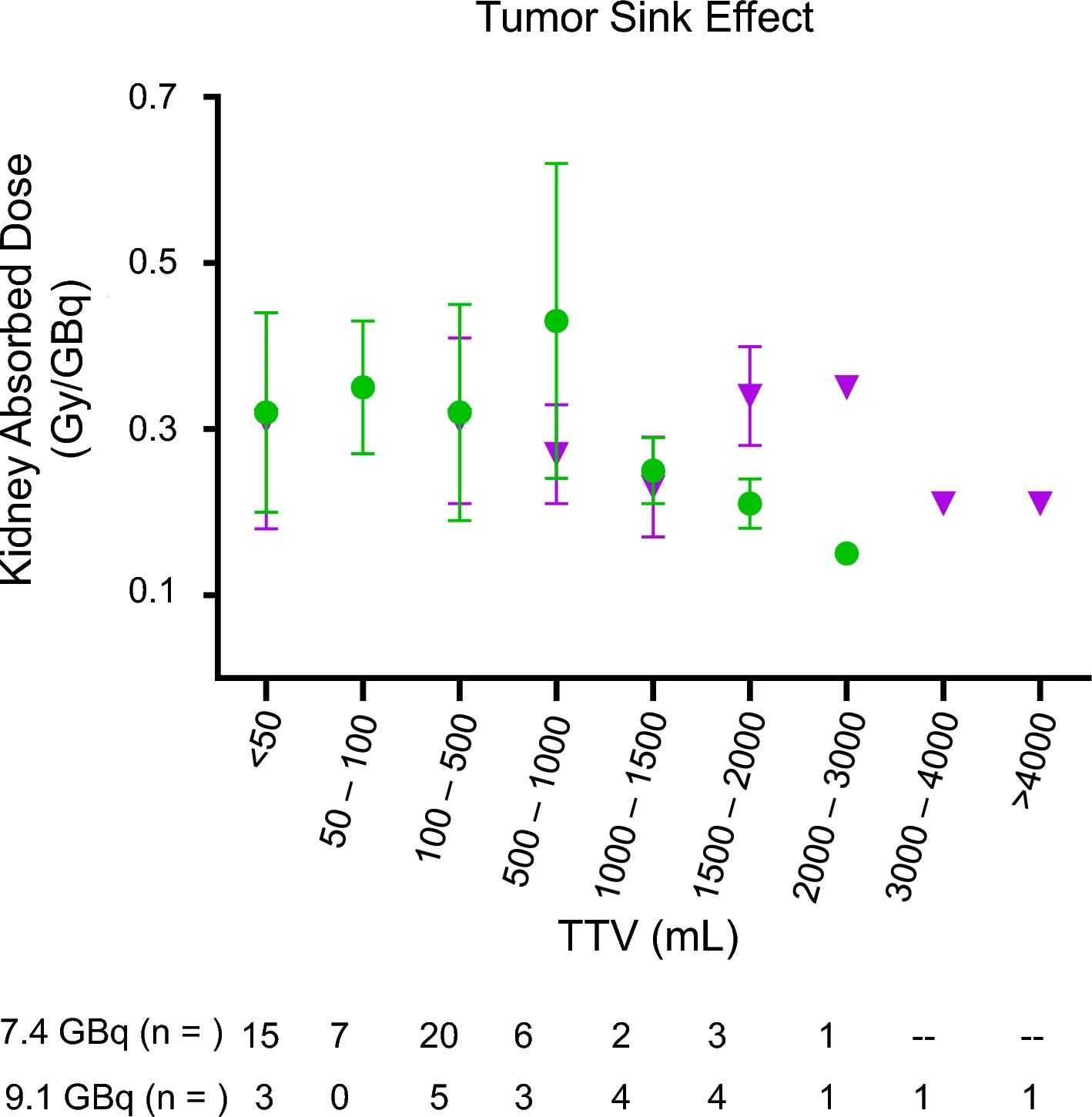
Lack of presence of the tumor sink effect in both injected activity regimens (7.4 GBq—Green Circles and 9.1 GBq—Purple Triangles)

The AD/GBq to the kidney from the two cohorts (7.4 GBq vs. 9.1 GBq) were comparable, 0.33 ± 0.13 Gy/GBq vs. 0.29 ± 0.08 Gy/GBq [*p* = 0.193]. No adverse renal side effects were found after the first cycle of therapy administered in either cohort. The AD/GBq to the kidney vs. TTV to investigate the tumor sink effect is shown in Fig. 6 (tumor sink effect with respect to tumors are shown in supplemental Fig. 2). No evidence of a clinically relevant tumor sink effect was found with respect to the kidney in patients with <2000 mL tumor volume; patients with substantially high TTV (> 2 L) lacks. A further comprehensive analysis including more patients with increased disease burdens is required prior to concluding.

## Discussion

Based on our AD findings, future activity escalation studies exceeding 9.1 GBq could be warranted as we found increased injected activities of [^177^Lu]Lu-PSMA-I&T exceeding 7.4 lead to similar ADs in both the kidney and tumors while not inducing acute toxicity. Other critical organs, including the bone marrow and salivary glands, were outside the scope of the present investigation. Consequently, caution is warranted when evaluating organ safety in studies employing increased administered activities, even though no kidney toxicity was observed. Individual analysis of the AD/GBq to the hottest and coldest lesion present in each patient was insignificant regarding injected activity. An increase in the AD/GBq to the kidney with reference to decreasing TTV was not found, indicating a lack of *tumor sink effect* in our patient population. Importantly, a statistically significant PSA response was found to be better in the 9.1 GBq cohort compared to 7.4 GBq.

Previously published methods have included tumor AD estimates from injected activities ranging from 3.6 to 7.5 GBq. While these studies have not shown drastic differences in the AD estimates to tumors various imaging protocols, segmentation methods, and AD estimates were employed. Here we compared the ADs from two different injected activities (standard 7.4 ± 0.1 vs. 9.1 ± 0.3 GBq) from the same center, employing the same AD estimation method to evaluate the influence on the AD estimates to the TTV and individual tumors. The reported similarity in kidney and tumor AD/GBq between cohorts reflects expected linear scaling within this increased activity range. The results do not provide evidence of saturation, redistribution, or nonlinear pharmacokinetics.

Additionally, tumor AD is governed by heterogeneous microenvironmental and histopathologic factors (e.g. perfusion variability, receptor density heterogeneity, necrosis, fibrosis, interstitial transport limitations, etc.). Given these are not evaluated at the tumor level, the absence of change in Gy/GBq from increased injected activity does not inform tumor biological dose–response, therefore changes are interpreted globally with respect to PSA.

We acknowledge limitations of the study are due to the segmentation approach (SUV-based thresholding) employed. Moreover, as stated, to reduce the AD calculation uncertainties owing to partial volume effects, misregistrations and patient movement during imaging, lesions smaller than 3 mL and rib lesions were excluded in the analysis, although the TTV and AD_TTV_ were practically unchanged. No further partial volume correction techniques were employed, and only monoexponiental functions were used to fit the time-activity data. Approaches to address this could be based on deconvolution-based image correction [34], correction factor based approaches [35, 36], or AI integration [37].

In our study, increasing the injected activity by 23% to 9.1 GBq had minimal impact on the AD/GBq to the kidneys and the whole tumor burden. Only bone lesions received a greater AD, statistically significant albeit in the 9.1 GBq cohort. Within the 7.4 GBq high TTV (500–2000 mL) cohort, bone lesions received significantly lower AD/GBq [*p* < *0.001*]. Lymph node lesions within this high TTV cohort showed no significance, but only included 5 patients in 7.4 GBq and 2 in 9.1 GBq cohorts respectively. Greater TTV did not influence individual tumor AD, regardless of injected activity or anatomical location (supplemental Fig. 1).

Evidence of acute toxicities remain uncommon in organs at risk [24], potentially providing evidence that higher administered activities are possible. Dosimetry was performed exclusively after the first treatment cycle, given that patients undergo multiple cycles at 6-week intervals with evolving tumor biology and organ kinetics, restriction to cycle 1 limits chronic toxicity assessment. Accurate computation of the AD [38] to the bone marrow [39, 40], salivary glands, and lacrimal glands [5, 41, 42], needs to be addressed prior to defining toxicity thresholds.

While the tumor sink effect was not seen, only 4 patients were present with over 2000 mL of disease preventing a sufficiently powered analysis. While current published data shows limited support of tumor sink using [^68^ Ga]Ga-PSMA-11 [43] and ^177^Lu-based PSMA-RPT [44, 45] we are currently investigating this further using a larger, bi-institutional cohort of patients.

Clinical trials like “[^177^Lu]Lu-DOTATATE Modified Delivery Based on Individualized Dosimetry” [LUMOD-ID (NCT06395402)] and “Intensified [^177^Lu]Lu-PSMA-617 Treatment for Patients With Metastatic Castrate-Resistant Prostate Cancer With Low PSMA Expressing Disease” [PSMA-Boost (NCT06526299)] are exploring if greater injected activities can possibly improve tumor control, and further define toxicity thresholds from RPT. It should be acknowledged that changing the injected activity is just one way of many to alter injection regimens and other prospective trials are looking at other aspects like time interval between consecutive cycles or total number of cycles [RIALTO (NCT06878664), [FLEX-MRT (NCT06216249)]).

## Conclusion

In this retrospective study patients administered 9.1 GBq opposed to 7.4 GBq, received comparable AD/GBq to the whole tumor burden, individual tumors, and kidneys. A statistically significant better PSA response was found in the 9.1 GBq cohort compared to 7.4 GBq. There was no evidence of the tumor sink effect in our study.

## Key points

### Question

What effect does a ~20% increase in the injected activity from 7.4 GBq [^177^Lu]Lu-PSMA-I&T have on the individual lesions, the total tumor volume, and the kidneys?.

### Pertinent findings

Our findings suggest that future dose escalation studies exceeding 9.1 GBq could be warranted with careful evaluation of other critical organs (e.g. bone marrow and salivary glands) as we found injected activities of [^177^Lu]Lu-PSMA-I&T at 7.4 and 9.1 GBq lead to similar AD/GBq in the kidney, whole tumor volume, and individual tumors. Individual analysis of the AD/GBq to the hottest and coldest lesion present in each patient also remained insignificant. An increase in the AD/GBq to the kidney with reference to decreasing TTV was not found, indicating a lack of *tumor sink effect* in our patient population. Importantly, a statistically significant PSA response was found to be better in the 9.1 GBq cohort compared to 7.4 GBq. No toxicities were found in the increased injected activity cohort following the first cycle of administration.

### Implications for patient care

Chronic toxicity can be prevented by individualized dosimetry, and it was found that increasing the administered activity from 7.4 to 9.1 GBq did not have any effect on the AD/GBq to the kidneys or tumors. Acute toxicity in this cohort of increased activity was not found. Modulation of the dose greater than 9.1 GBq could be warranted with further prospective clinical trials encompassing similar patient cohorts and standardized imaging protocols.

## Data availability and materials

Please contact the corresponding author.

## Supplementary Information

Supplementary Material 1.

## Abbreviations

PSMA: Prostate membrane specific antigen
AD: Absorbed dose
TTV: Total tumor volume
RPT: Radiopharmaceutical therapy
