# Supplementary material for: Influence of increased injected activities up 9.0 GBq [177Lu]Lu-PSMA-I&T on absorbed doses to lesions and kidneys
Source: EJNMMI Phys. 2026 Sep 11;13:92. doi: 10.1186/s40658-026-00932-x (PMC13569807; doi:10.1186/s40658-026-00932-x)
Supplement: Supplementary file 1 [file 40658_2026_932_MOESM1_ESM.docx]

Table 1: TTV as defined in segmentation on SPECT using a thresholding approach described in a prior publication. Recall AD_TTV_ is defined as the TTV used in the absorbed dose calculation which removed the small volume, and rib lesions as discussed in the methods section. *%D: Percent Difference

|  | 7.4 GBq | | 9.1 GBq | | P – Value |
| --- | --- | --- | --- | --- | --- |
|  | Average ± SD | Median  [Range] | Average ± SD | Median  [Range] |  |
| TTV  (mL) | 396.1 ± 563.1 | 192.4  [5.4 – 2334.5] | 1409.4 ± 1294.7 | 1075.5  [31.0 – 4711.0] | <0.002 |
| AD_TTV_  (mL) | 368.9 ± 521.2 | 189.8  [4.2 – 2182.5] | 1164.5 ± 1157 | 947.3  [21.7 – 4308.9] | <0.005 |
| TTV - %D* (%) | -11.2 ± 12.1 | -7.3  [0.0 – -53.7] | -23.6 ± 21.9 | -17.4  [0.0 – -72.1] | -- |
| 100 – 380  (mL) | 244.7 ± 78.9 | 246.1  [121.6 – 354.3] | 253.6 ± 114.0 | 319.8  [100.0 – 354.3] | 0.839 |
| 500 – 2000  (mL) | 1074 ± 532.7 | 963.1  [501.5 – 1830.2] | 1251.7 ± 436.9 | 1191  [597.6 – 1869.0] | 0.402 |

The row “TTV - %D” represents the present difference between the two approaches. To harmonize the data and compare like groups we created a sub-analysis by identifying groups of patients based on their total tumor volumes used in the absorbed dose calculation (i) 100 – 380 mL and (ii) 500 – 2000 mL.

Table 2: Average Absorbed Dose to Whole Tumor Volume

|  | 7.4 GBq | | 9.1 GBq | | P – Value |
| --- | --- | --- | --- | --- | --- |
|  | Average ± SD | Median [Range] | Average ± SD | Median  [Range] |  |
| *7.4 GBq n = 54 vs. 9.1 GBq n = 22* | | | | | |
| **AD_TTV_**  **(mL)** | 6.7 ± 3.9 | 5.7  [1.9 – 18.9] | 7.7 ± 3.8 | 7.0  [2.5 – 18.3] | 0.302 |
| *7.4 GBq n = 19 vs. 9.1 GBq n = 5* | | | | | |
| **100 – 380**  **(mL)** | 7.0 ± 4.3 | 5.7  [1.3 – 18.9] | 9.8 ± 6.4 | 6.3  [3.5 – 18.3] | 0.770 |
| *7.4 GBq n = 11 vs. 9.1 GBq n = 11* | | | | | |
| **500 – 2000**  **(mL)** | 7.1 ± 4.2 | 7.1  [2.9 – 18.0] | 7.6 ± 2.5 | 7.9  [4.0 – 11.8] | 0.250 |

Table 3: Average Absorbed Dose to Individual Tumors

| Location | 7.4 GBq | | | | 9.1 GBq | | | | P – Value |
| --- | --- | --- | --- | --- | --- | --- | --- | --- | --- |
|  | Number of Patients | Total Number of Lesions | Average ± SD | Median | Number of Patients | Total Number of Lesions | Average ± SD | Median |  |
| **AD_TTV_ - 7.4 GBq n = 54 vs. 9.1 GBq n = 22** | | | | | | | | | |
| Bone | 46 | 330 | 5.4 ± 3.4 | 4.7 | 19 | 183 | 7.1 ± 3.8 | 6.7 | <0.0001 |
| LN | 22 | 71 | 8.6 ± 5.2 | 6.8 | 6 | 43 | 6.7 ± 4.4 | 4.9 | 0.051 |
| Liver | 2 | 4 | 9.8 ± 5.5 | 10.1 | 1 | 2 | 7.1 ± 1.0 | 7.1 | 0.548 |
| **100 – 380 (mL) - 7.4 GBq n = 19 vs. 9.1 GBq n = 5** | | | | | | | | | |
| Bone | 17 | 150 | 5.7 ± 3.8 | 5.0 | 4 | 15 | 14.3 ± 6.4 | 14.8 | 0.048 |
| LN | 11 | 40 | 8.1 ± 5.2 | 6.2 | 4 | 21 | 5.31 ± 1.0 | 0.53 | 0.834 |
| Liver | 1 | 1 | 3.64 | -- | 1 | 2 | 7.1 ± 3.9 | 7.1 | -- |
| **500 – 2000 (mL) 7.4 GBq n = 11 vs. 9.1 GBq n = 11** | | | | | | | | | |
| Bone | 11 | 108 | 5.2 ± 2.9 | 4.8 | 10 | 134 | 6.6 ± 2.8 | 6.4 | <0.0001 |
| LN | 5 | 18 | 8.8 ± 9.9 | 5.4 | 2 | 20 | 7.8 ± 4.8 | 6.6 | 0.215 |
| Liver | 1 | 3 | 13.5 ± 11.9 | 4.5 | -- | -- | -- | -- | -- |

Table 4: Average Volume (mL) of Individual Tumors

| Location | 7.4 GBq | | | | 9.1 GBq | | | | P – Value |
| --- | --- | --- | --- | --- | --- | --- | --- | --- | --- |
|  | Number of Patients | Total Number of Lesions | Average ± SD | Median | Number of Patients | Total Number of Lesions | Average ± SD | Median |  |
| **AD_TTV_ - 7.4 GBq n = 54 vs. 9.1 GBq n = 22** | | | | | | | | | |
| Bone | 46 | 330 | 49.8 ± 160.8 | 10.8 | 19 | 183 | 127.0 ± 399.3 | 15.5 | **0.013** |
| LN | 22 | 71 | 46.1 ± 81.5 | 13.3 | 6 | 43 | 48.8 ± 110.1 | 10.3 | 0.890 |
| Liver | 2 | 4 | 50.9 ± 54.1 | 42.1 | 1 | 2 | 136.9 ± 143.2 | 136.9 | -- |
| **100 – 380 (mL) - 7.4 GBq n = 19 vs. 9.1 GBq n = 5** | | | | | | | | | |
| Bone | 17 | 150 | 24.4 ± 36.2 | 5.0 | 4 | 15 | 13.2 ± 16.2 | 7.0 | -- |
| LN | 11 | 40 | 24.6 ± 35.7 | 6.2 | 4 | 21 | 38.0 ± 56.3 | 13.3 | -- |
| Liver | 1 | 1 | 5.7 | -- | 1 | 2 | 136.9 ± 143.2 | 136.9 | -- |
| **500 – 2000 (mL) 7.4 GBq n = 11 vs. 9.1 GBq n = 11** | | | | | | | | | |
| Bone | 11 | 108 | 88.7 ± 189.8 | 19.7 | 10 | 134 | 93.2 ± 225.4 | 16.1 | -- |
| LN | 5 | 18 | 113.1 ± 133.4 | 47.8 | 2 | 20 | 63.9 ± 151.4 | 9.9 | -- |
| Liver | 1 | 3 | 66.0 ± 54.9 | 78.3 | -- | -- | -- | -- | -- |

Hottest (Table 5) and Coldest (Table 6) Lesion Present in each patient cohort. No statistical significance was found between the two injection activity regimens with respect to the lesions that received the highest (hottest) and lowest (coldest) AD.

Table 5:

| **Hottest** | 7.4 GBq | | | 9.1 GBq | | | P-Value |  |
| --- | --- | --- | --- | --- | --- | --- | --- | --- |
|  | Average ± SD | Median | Range | Average ± SD | Median | Range |  |  |
| 7.4 GBq n = 47 vs. 9.1 GBq n = 22  [Included Lesions] 7.4 GBq: 34 Bone, 12 LN, 1 Liver vs. 9.1 GBq: 15 Bone, 6 LN, 1 Liver | | | | | | | | |
| AD_TTV_ (Gy) | 9.5 ± 5.4 | 8.3 | [1.9 – 22.2] | 10.7 ± 5.6 | 9.2 | [2.9 – 25.7] | 0.428 |  |
| Size (mL) | 40.9 ± 66.9 | 16.5 | [3.0 – 314.0] | 289.2 ± 707.3 | 19.1 | [3.6 – 2856.2] | 0.115 |  |
| 7.4 GBq n = 19 vs. 9.1 GBq n = 5 [Included Lesions] 7.4 GBq: 12 Bone, 7 LN vs. 9.1 GBq: 1 Bone, 3 LN, 1 Liver | | | | | | | |  |
| AD_TTV_ (Gy)  100 – 380mL | 10.7 ± 6.3 | 8.7 | [1.9 – 22.2] | 13.3 ± 7.8 | 8.9 | [7.8 – 25.7] | 0.524 |  |
| Size (mL) | 27.8 ± 36.7 | 14.6 | [4.8 – 128.8] | 36.4 ± 37.6 | 27.3 | [9.0 – 100.4] | 0.662 |  |
| 7.4 GBq n = 11 vs. 9.1 GBq n = 11  [Included Lesions] 7.4 GBq: 7 Bone, 3 LN, 1 Liver vs. 9.1 GBq: 9 Bone, 2 LN | | | | | | | |  |
| AD_TTV_ (Gy)  0.5 – 2 L | 9.7 ± 4.5 | 9.2 | [5.2 – 20.1] | 11.3 ± 4.8 | 9.5 | [6.3 – 22.2] | 0.414 |  |
| Size (mL) | 103.5 ± 110.5 | 50.8 | [8.5 – 314.3] | 134.2 ± 328.3 | 18.2 | [6.9 – 1111.9] | 0.774 |  |

Table 6:

| **Coldest** | 7.4 GBq | | | 9.1 GBq | | | P-Value |  |
| --- | --- | --- | --- | --- | --- | --- | --- | --- |
|  | Average ± SD | Median | Range | Average ± SD | Median | Range |  |  |
| 7.4 GBq n = 47 vs. 9.1 GBq n = 22  7.4 GBq: 40 Bone, 7 LN vs. 9.1 GBq: 17 Bone, 5 LN | | | | | | | | |
| AD_TTV_  (Gy) | 3.7 ± 2.4 | 3.1 | [0.3 – 14.0] | 3.6 ± 1.5 | 3.3 | [1.8 – 8.1] | 0.917 |  |
| Size (mL) | 16.8 ± 34.8 | 6.4 | [3.0 – 186.6] | 8.6 ± 8.6 | 6.0 | [2.9 – 43.0] | 0.135 |  |
| 7.4 GBq n = 19 vs. 9.1 GBq n = 5  7.4 GBq: 16 Bone, 3 LN vs. 9.1 GBq: 2 Bone, 3 LN | | | | | | | |  |
| AD_TTV_  (Gy)  100 – 380mL | 3.7 ± 2.0 | 3.4 | [0.3 – 8.4] | 4.5 ± 2.5 | 3.6 | [1.8 – 8.1] | 0.563 |  |
| Size (mL) | 16.8 ± 41.3 | 6.4 | [3.3 – 186.6] | 5.1 ± 1.7 | 5.8 | [3.1 – 6.9] | 0.234 |  |
| 7.4 GBq n = 11 vs. 9.1 GBq n = 11  7.4 GBq: 9 Bone, 2 LN vs. 9.1 GBq: 9 Bone, 2 LN | | | | | | | |  |
| AD_TTV_  (Gy)  0.5 – 2 L | 4.0 ± 3.4 | 3.1 | [1.8 – 14.0] | 3.3 ± 1.0 | 2.7 | [2.2 – 5.2] | 0.530 |  |
| Size (mL) | 24.0 ± 45.7 | 5.5 | [3.8 – 150.7] | 10.8 ± 11.6 | 6.2 | [2.7 – 43.0] | 0.373 |  |

**Figure 1:** Tumor AD per injected activity and anatomical location compared to stratified TTV. Left (column 1 – green) shows 7.4 GBq cohort while right (column 2 – purple) shows 9.1 GBq cohort. Based on TTV, lesions stratified by anatomical location did not have a significantly higher AD except in the case of bone lesions in the 9.1 GBq cohort.

**Figure 2:** X-axis bins patients based on TTV. Increased TTV does not influence the AD_TTV_

(top), hottest lesion (middle), or coldest lesions (bottom) present in a patient.
